# Direct Scaling of Measure on Vortex Shedding through a Flapping Flag Device in the Open Channel around a Cylinder at *Re*∼10^3^: Taylor’s Law Approach

**DOI:** 10.3390/s21051871

**Published:** 2021-03-08

**Authors:** Samuele De Bartolo, Massimo De Vittorio, Antonio Francone, Francesco Guido, Elisa Leone, Vincenzo Mariano Mastronardi, Andrea Notaro, Giuseppe Roberto Tomasicchio

**Affiliations:** 1 Department of Engineering for Innovation, EUropean Maritime Environmental Research (EUMER), University of Salento, Via per Monteroni, 73100 Lecce, Italy; samuele.debartolo@unisalento.it (S.D.B.); massimo.devittorio@unisalento.it (M.D.V.); elisa.leone@unisalento.it (E.L.); giuseppe.tomasicchio@unisalento.it (G.R.T.); 2 Center for Biomolecular Nanotechnologies (CBN), Italian Institute of Technology (IIT), Via Barsanti 14, 73100 Lecce, Italy; francesco.guido@iit.it (F.G.); vincenzo.mastronardi@iit.it (V.M.M.); 3 Department of Civil, Environmental, Land, Construction and Chemistry Engineering, Polytechnic of Bari, Via Edoardo Orabona 4, 70125 Bari, Italy; antonio.francone@poliba.it; 4 School of Advanced Studies ISUFI, University of Salento, Via per Monteroni, 73100 Lecce, Italy

**Keywords:** sensor technologies, sensor systems for water flow, hydrodynamics monitoring of rivers, hydraulics, MEMS, fluid–structure interactions

## Abstract

The problem of vortex shedding, which occurs when an obstacle is placed in a regular flow, is governed by Reynolds and Strouhal numbers, known by dimensional analysis. The present work aims to propose a thin films-based device, consisting of an elastic piezoelectric flapping flag clamped at one end, in order to determine the frequency of vortex shedding downstream an obstacle for a flow field at Reynolds number $Re∼10^{3}$ in the open channel. For these values, Strouhal number obtained in such way is in accordance with the results known in literature. Moreover, the development of the voltage over time, generated by the flapping flag under the load due to flow field, shows a highly fluctuating behavior and satisfies Taylor’s law, observed in several complex systems. This provided useful information about the flow field through the constitutive law of the device.

## 1. Introduction

In the previous years, the development of fluid-dynamic measuring instruments has been steadily increasing. Most flows encountered in practice are turbulent, and a disk or a short cylinder placed in the flow coaxially sheds vortices. It is observed that these vortices are shed periodically, and the shedding frequency is proportional to the average flow velocity. This suggests that the flow rate can be determined by generating vortices in the flow by placing an obstruction along the flow and measuring the shedding frequency [1]. The flow measurement devices that work on this principle are called vortex flowmeters. They consist of a sharp-edged bluff body (strut) placed in the flow that serves as the vortex generator, and a detector (such as a pressure transducer that records the oscillation in pressure) placed within a short distance downstream on the inner surface of the casing aiming to measure the shedding frequency. The frequency of vortex shedding is proportional to the average velocity over a wide range of Reynolds numbers, and vortex flowmeters operate reliably and accurately at Reynolds numbers from $10^{4}$ to $10^{7}$. The advantage of vortex flowmeter is that it has no moving parts and thus it is inherently reliable, versatile, and very accurate, but it obstructs flow and thus causes considerable head. Thermal anemometers, introduced in the late 1950s, can take thousands of velocity measurements per second with excellent spatial and temporal resolution, and thus they can be used for studying details of fluctuations in the turbulent flow. A thermal anemometer is called a hot-wire anemometer if the sensing element is a wire. The hot-wire anemometer is characterized by its very small sensor wire—usually a few microns in diameter and a couple of millimeters in length. The operating principle of a constant-temperature anemometer (CTA), which is the most common type, is as follows. The sensor is electrically heated to a specified temperature (typically about 200 ${}^{\circ }$C), and it tends to cool as it loses heat to the surrounding flowing fluid, but electronic controls maintain the sensor at a constant temperature by varying the electric current (which is done by varying the voltage) as needed. The higher the flow velocity, the higher the rate of heat transfer from the sensor, and thus the larger the voltage that needs to be applied across the sensor to maintain it at constant temperature. There is a close correlation between the flow velocity and voltage, and the flow velocity can be determined by measuring the voltage applied by an amplifier or the electric current passing through the sensor. Unlike thermal anemometry, Laser Doppler velocimetry (LDV) [2] involves no probes or wires inserted into the flow, and thus it is a nonintrusive method. Like thermal anemometry, it can accurately measure velocity at a very small volume, and thus it can also be used to study the details of flow at a locality, including turbulent fluctuations, and it can be traversed through the entire flow field without intrusion. The operating principle of LDV is based on sending a highly coherent monochromatic (all waves are in phase and at the same wavelength) light beam toward the target, collecting the light reflected by small particles in the target area, determining the change in frequency of the reflected radiation due to Doppler effect, and relating this frequency shift to the flow velocity of the fluid at the target area. The particle image velocimetry (PIV) [3] is a double-pulsed laser technique used to measure the instantaneous velocity distribution in a plane of flow by photographically determining the displacement of particles in the plane during a very short time interval. Unlike methods like hot-wire anemometry and LDV that measure velocity at a point, PIV provides velocity values simultaneously throughout an entire cross section, and thus it is a whole-field technique. PIV combines the accuracy of LDV with the capability of flow visualization and provides instantaneous flow field mapping.

As it is well known, vortex shedding phenomenon is governed by a Strouhal number that represents the ratio of inertial forces due to the local acceleration of the flow to the inertial forces due to the convective acceleration [4]. Several studies aimed to define the relationship between Strouhal and Reynolds numbers have followed each other through the years. Originally [5], measurements on a large circular cylinder in a pressurized wind tunnel at Reynolds numbers from $10^{6}$ to $10^{7}$ have revealed a high Reynolds number transition in which the drag coefficient increases from its low supercritical value to a value 0.7 at $Re=3.5\cdot 10^{6}$ and then becomes constant. In addition, for $Re>3.5\cdot 10^{6}$, a definite vortex shedding occurs, with Strouhal number 0.27. According to the experiment of Bearman [6], vortex shedding frequencies were measured by using a model with splitter plates, in which an oscilloscope displaying the fluctuating voltage of a hot wire gave an accurate picture of the velocity fluctuation at the hot-wire probe. In the same way, a constant value of St=0.178 was found by Griffin [7] for subcritical wake Reynolds numbers from 700 to $5\cdot 10^{4}$. Okajima [8] determined Strohual numbers of rectangular cylinders as a function of Reynolds number in the range between 70 and $2\cdot 10^{4}$, using an open-jet small wind tunnel and a towing-type water tank. A sudden discontinuity in the Strouhal-number curves was noted for a ratio width/height of the cylinder in the range between 2 and 3, highlighting the strong dependence on dimensional characteristics. A few years later, Gonçalves and Vieira [9] performed a set of experiments in a pilot vertical low turbulence hydrodynamic tunnel, where the emission of liquid dye, directly in non-perturbed flow by means of a long hypodermic needle, has been utilized to create the flow image captured by an A3 CCD high-resolution video camera in order to determinate Strouhal number for Reynolds up to 600, as a result of processing. Taylor et al. [10] have shown that birds, bats, and insects in a cruising flight flap their wings within a narrow range of Strohual number 0.2<St<0.4, since the animals tune their frequency to achieve maximum propulsive efficiency. More recently, Eloy [11] predicted the optimal Strohual number for swimming animals by the use of Lighthill’s large-amplitude elongated-body theory. With the spread of optical methods, the particle image velocimetry (PIV) technique has been used by Shi et al. [12] to visualize the vortex shedding processes from parallel-plate thermoacoustic stacks in oscillatory flow conditions within an acoustic cycle, phase-by-phase, in particular during the part of the cycle when the fluid flows out of the stack—selected cases are shown for comparisons with hot-wire measurements. The wake characteristics and the vortex shedding process in the flow past a circular cylinder may also be studied through Large eddy simulation (LES), which is a mathematical model for turbulence used in computational fluid dynamics, as made by Rodriguez et al. [13] for the range of Reynolds numbers $2.5\cdot 10^{5}<Re<8.5\cdot 10^{5}$.

The innovative element introduced in this work is the use of a thin films-based piezoelectric cantilever, that acts as a flexible piezoelectric transducer, to determine the frequency of the vortex shedding and consequently the Strouhal number St associated with it, for a particular range of flow field in open channel, alternatively to the instruments used in fluid dynamics previously mentioned. In the context of fluid dynamics, the microcantilever has already found application as a rheological sensor to measure the properties of Newtonian and non-Newtonian fluids in real time [14] and for sensing of both the flow rate and the flow direction [15]. On the other hand, the piezoelectric cantilever is also used to harvest energy [16,17,18,19,20,21,22,23,24], in the Internet-of-Things field [20,25,26], to realize piezoelectrochemical hydrogen production [27], for biochemical sensing, parallel multicantilever Atomic Force Microscopy (AFM) measurements and nanometer range manipulation [28]. The strengths of this device are low cost, easily manufactured; although intrusive, it is well suited for small scale models and can be used as a self-powering sensor. The mechanical and electrical characteristics of the device and a general description of the experimental setup will be presented in Section 2.1. Particular attention has been paid to the danger of the fatigue stress and subsequent failure [29]. Moreover, we must verify that the frequency of the release is not too close to the natural frequency of vibration of the structure in order to avoid the phenomenon of resonance. To better understand the aggregation processes with which the signal acquired by the device evolves, the correlation function has been defined Section 3.1. In Section 3.2, a comparison with the results known in literature regarding the relationship between Strouhal and Reynolds numbers will be carried out. Subsequently, a second order theory based on mean and variance, described in Section 3.3, has been applied to experimental data, in terms of output voltage, to recognize a scaling power law, evaluate the development of scale exponent over time, and characterize some aspects in the context of the vortex shedding, such as statistical distribution and the ergodicity, through some relationships that connect the voltage fluctuations with the fluid dynamics variables, such as velocity and pressure. Finally, in Section 4, a critical discussion of results is undertaken.

## 2. Materials and Methods

### 2.1. Thin Films-Based Device and Experimental Setup

**Figure 1:**
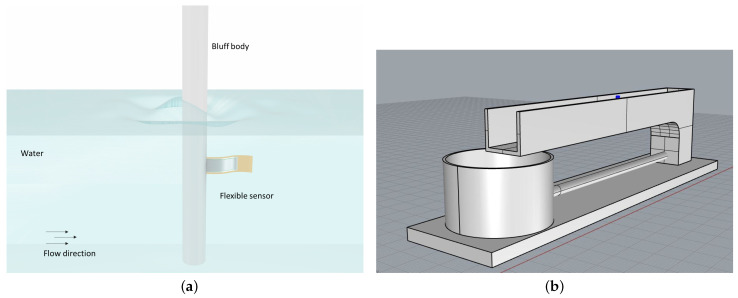
The sensor, mounted downstream of the bluff body, is affected by the vortexes and oscillates generating alternating voltage (**a**). 3d view of the channel flume site in EUMER Lab of Unversity of Salento (**b**).

The thin-films-based device, previously mentioned, consists of a flag, clamped at one end to a cylinder of diameter D = 2.00 cm [30,31], which oscillates in a media flow, as shown in Figure 1a, and converts kinetic energy into electrical energy [23].

**Figure 2:**
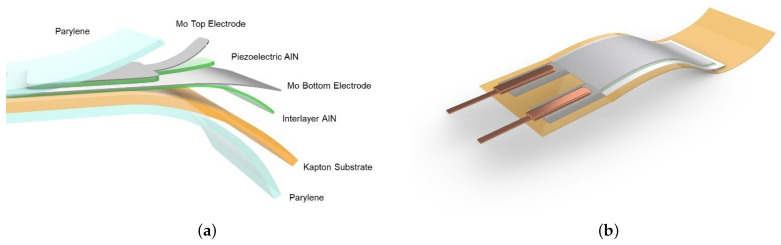
The flexible structure of the piezoelectric devices was optimized to guarantee high flexibility and sensitivity. In addition all the materials used for its fabrication are completely biocompatible, suitable for environmental use (**a**). The 3D sketch highlights the extremely compactness of the transducer (**b**).

The experimental set-up is reported in Figure 1b showing the channel flume at a site in EUMER Lab at University of Salento, where there is a water flow in steady conditions with density and kinematic viscosity equal to ρ = 1000 kg m${}^{-3}$ and ν = $10^{-6}$ m${}^{2}$ s${}^{-1}$ at the temperature of 25 °C, respectively. The flag device acts as a flexible piezoelectric transducer. It is mainly based on a polyimide substrate (Kapton 100HN, 25 μm of thickness), on which a multilayered structure was grown and microfabricated in order to guarantee high sensitivity and flexibility. A detailed sketch is reported in Figure 2.

**Figure 3:**
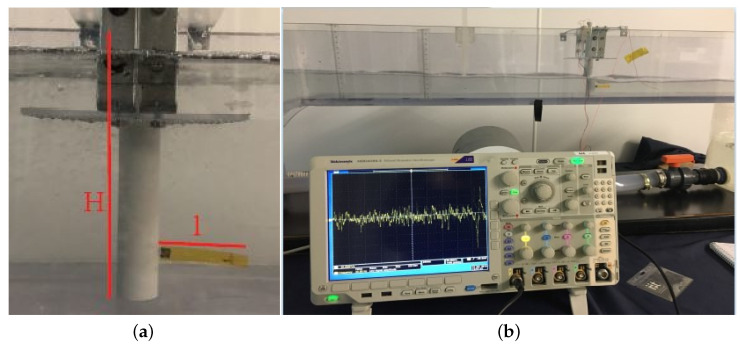
Flapping flag clamped at the cylinder (**a**) and Oscilloscope Tektronix MSO2000B (**b**).

The multi-layered thin films structure consists of a piezoelectric layer of Aluminum Nitride (AlN, 1 μm thick) sandwiched between two Molybdenum films (Mo, 200 nm of thickness) acting as electrodes. AlN is a dielectric material presenting interesting electrical and a natural piezoelectricity due to its crystal symmetry [16,20,21,28]. Since the device will be employed as an underwater sensor, it needs to be fully electrically insulated. For this reason, a thin film of Parylene-C (1.5–2.5 μm) is uniformly deposited, at room temperature. This insulating layer covers the final device in a conformal way, making it perfectly waterproof. The dimensions of the active piezoelectric area are 1.7 × 0.4 cm${}^{2}$ and it is positioned at the hinge of the flapping structure, where the mechanical stress is higher during the flapping-induced oscillations. To fabricate the flexible devices, standard microfabrication procedures as photolithography, wet and dry etching processes were performed. In addition, a CNC laser cutter was used to define the elastic substrate geometry. The last step of the fabrication process consisted of the mechanical crimping of the electrodes in order to connect the device, allowing the reading and post processing of the electrical voltage signal generated by the piezoelectric film. The part of the device with the electrical connections was then embedded into the cylindrical bluff body, keeping still the connections waterproof. The voltage fluctuations v(t) generated by mode shapes and deflections of the flapping flag, shown in Figure 3a, due to the flow field were then recorded by an oscilloscope (Tektronix, MSO2000B) (see Figure 3b).

**Figure 4:**
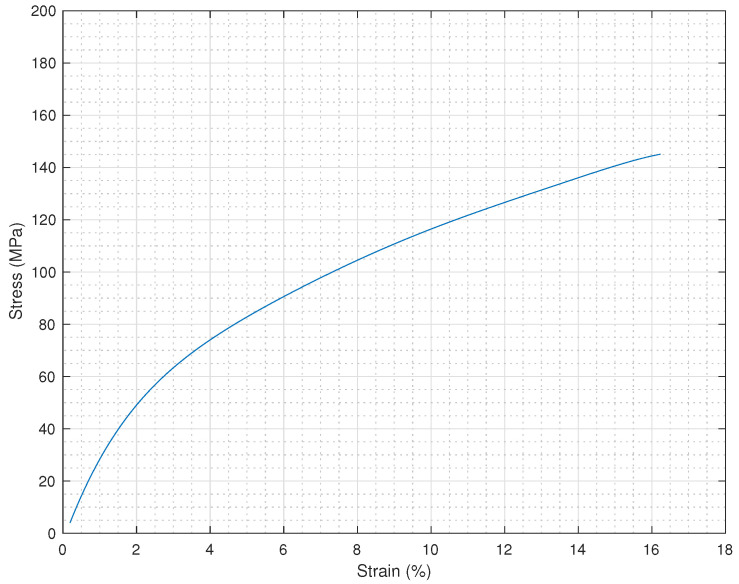
Stress–strain relationship of the composite flag obtained by Dynamic Mechanical Analysis (DMA).

Several measurements were performed in order to find a constitutive law and all the stress–strain curves present the same shape, with an initial linear portion and then a non-linear region. These curves result from Dynamic Mechanical Analysis (DMA) of the AlN-based flexible flag. The Young’s modulus was determined as the slope of the first portion of the stress-strain curves and calculated as an average value of several measurements. Dynamic mechanical measurements were performed in controlled force mode (force rate 1 N/min) with a Q800 instrument (TA Instruments). The Figure 4 reports the average curve obtained by DMA measurements, where a Young’s Modulus *E* = 2.5 ± 0.3 GPa was extrapolated.

The output voltage of the flexible transducer is directly related to the stress provoked on the thin films structure by elastic deformations. Since the extremely flexible structure is subjected to the vortices shedding downstream to an obstacle of cylindrical shape, the deformations are very large; we would like to stress that the transducer is composed of two parts: an active part, including the piezoelectric layer, and a pure elastic part consisting only of the Kapton layer. The elastic part acts as a “fluid fluctuations collector” and the order of its deformations is about one centimeter; the active part, close to the hinge of the structure is subjected to lower deformations, with the order of its deformations of few millimeters. For these reasons, the structure can be approximated to a simple cantilever beam if, and only if, we consider its piezoelectric part. For the aim of this manuscript, this approximation is adequate to guarantee the relation between the output generated voltage and the vortices shedding.

### 2.2. Strohual and Reynolds Numbers Relationship

**Figure 5:**
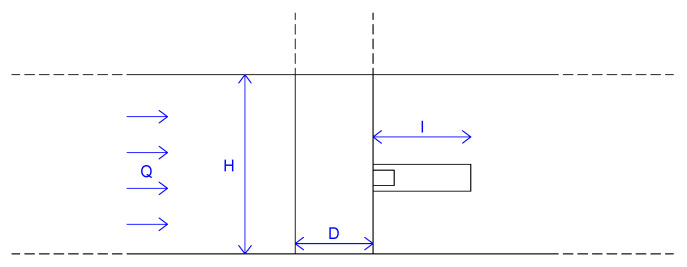
Longitudinal section of the experimental setup with the flag of length *l* clamped at the cylinder of diameter *D*, inlet flow rate *Q*, and height of flow *H* in the open channel.

The variables involved in this problem are the following: frequency of vortex shedding $f_{r}$, the height of flow in the open channel *H*, the diameter of cylinder *D*, the length of flag *l*, bulk velocity *u*, the kinematic viscosity ν. Since the reference dimensions are two (length and time), according to Buckingham Theorem we have four independent dimensionless groups, among them Strouhal and Reynolds numbers. Therefore, we need to establish the values of Reynolds and Strouhal numbers for the flow field in which this experiment occurs. The assumptions used in the present study are: the flow is two-dimensional and the fluid satisfies the incompressible Newtonian fluid assumptions, with Reynolds number variable between 2000 and 8000, the piezoelectric constitutive material model is linear, and the beam is thin and obeys the Euler–Bernoulli formulation [32], hence deflections are small when compared to the beam dimensions [14]. As stated in Section 1, there are two non-dimensional parameters that will govern the flow around a circular cylinder in a uniform shear flow [33]. Reynolds number is the ratio of inertial forces to viscous forces and is a convenient parameter for predicting if a flow condition will be laminar or turbulent. It can be interpreted that when the viscous forces are dominant (slow flow, low Re) they are sufficient enough to keep all the fluid particles in line, then the flow is laminar. Even very low Re indicates viscous creeping motion, where inertia effects are negligible. When the inertial forces dominate over the viscous forces (when the fluid is flowing faster and Re is larger) then the flow is turbulent. Reynolds number is defined as:

(1) $$Re=\frac{u\;D}{\nu }$$

where *u* is the bulk velocity, *D* is the diameter of cylinder, and ν is the kinematic velocity of the fluid [34]. Instead, Strouhal number St is given by [35]:

(2) $$St=\frac{f_{r}\;D}{u}$$

where $f_{r}$ is the frequency of vortex shedding from one side of the cylinder (in s${}^{-1}$), *u* is the bulk velocity, and *D* is the diameter of cylinder. Dimensional analysis shows that Strohual number may be expressed as a function of other dimensionless groups: St= *f(H/D, l/D, 1/Re)*, hence it is important to understand the relationship with Reynolds number. A number of empirical and semi-empirical formulae for the St–Re relationship over certain ranges of Re were proposed [36], for example in [37,38,39,40,41,42]. Once a steady inlet flow rate *Q* was set equal to 2.16 $l\;s^{-1}$, several configurations have been studied by varying the length of the flag *l* and the height of the flow *H* in the open channel. According to the continuity equation, in the stationary case, bulk velocity is given by u=Q/(BH) in which *B* = 0.16 m is the width of the channel flume, whereas *H* is the height of the flow in the open channel set by means of some hydraulic systems, such as sluice gates, reducing the flow section upstream of the cylinder (see Figure 5). Knowing the bulk velocity *u*, Reynolds number Re is obtained by Equation (1).

The period of “von Kármán’s vortex street”, due to the bluff body which disturbs the regular flow and generates turbulence, is defined as follows [23]:

(3) $$\lambda =\frac{0.85\;u}{St\;\frac{u}{D}}=4.25\;D$$

since the whirls released from the bluff body travel with velocity of about 85% of the flow velocity *v*. One basic requirement was that the cantilevers should oscillate only with their first harmonic mode. Therefore, the length of the cantilever L is limited to the half of the period of the “von Kármán’s” vortex street λ [17]. For the ratio L to D, one can find L/D = 2.125 [22]. According to Pobering and Schwesinger [22], the pressure difference Δ*p* can be calculated assuming that the rotational flow velocity of the whirls $u_{r}$ is about 1/3 of the flow velocity *u* of the undisturbed fluid. Furthermore, taking into account that a gap exists between two whirls on the opposite side of the cantilever, one can achieve for Δ*p*, the following relationship [22] based on Bernoulli’s Theorem considerations:

(4) $$\Delta p=\frac{\rho }{2}\left({\left(u-\frac{u}{3}\right)}^{2}-u^{2}\right)$$

where ρ and *u* have been previously defined. Hence, the proposed sensor is also able to measure the difference of pressure from each side of the flag by means the following relationship that is derived from Pobering and Schwesinger [22] for a cantilever similar to our device:

(5) $$v_{peak}=K\Delta p\frac{d_{31}}{e_{0}e_{r}}\frac{l^{2}}{T_{pzt}}$$

in which $v_{peak}$ is the peak voltage developing on the electrodes during the deflection, $d_{31}$ the piezoelectric strain constant, $e_{r}$ the relative dielectric constant, $e_{0}$ the dielectric constant in vacuum, $T_{pzt}$ the thickness of piezoelectric material, *l* the length of the cantilever, and *K* is a constant that will be validated later in Section 3.2 according to our configurations. As discussed in Section 1, high vibrations on the structure can damage it and for a long-term analysis, the fatigue stress and subsequent fatigue failure could be significant. For this purpose, preliminary Finite Elements Method (FEM) simulations were performed in order to evaluate the mechanical behavior of the flexible transducers and its fundamental resonance frequencies. The natural frequency of vibration $f_{1}$ depends on bending stiffness, Young’s modulus, length, width and mass of the cantilever beam [14,19].

**Figure 6:**
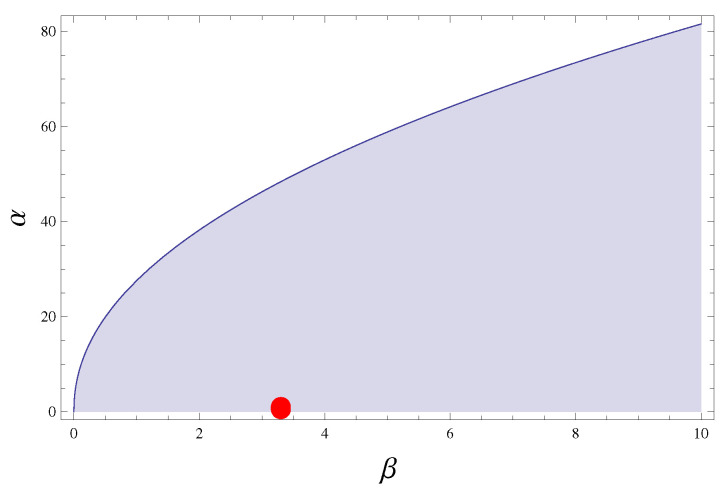
Domain of stability for the flapping flag marked by the shaded zone [30]. The red point represents our experimental data.

In particular, replicating the dimensions and the shapes of the sensors under test, we found the values of natural frequency of vibration $f_{1}$, for each length cantilever analyzed, listed in Table 1. To ensure the correct use of the sensor, it is necessary to check that the studied velocities are below the critical value. For this purpose, with the reference to the studies carried out previously [30], we demonstrate that experiments fall into the stability area (see Figure 6) defined as function of two non-dimensional quantities $\alpha =u/u_{b}$ and $\beta =\rho_{s}/\rho$, where $\rho_{s}$ is the density of the flag equal to 3200 kg m${}^{-3}$, ρ is the density of water, and $u_{b}=\sqrt{EI/\rho_{s}}$ a characteristic bending wave velocity (I is the moment of inertia of cross-section of the cantilever with reference to the neutral axis and *E* is the Young’s Modulus determined in Section 2.1).

### 2.3. Direct Scaling Analysis on the Voltage Fluctuations: Taylor’s Law Approach

Then, our efforts have been focused on identifying a scaling power law based on experimental data. *Taylor’s law*, also known as fluctuation scaling in physics [43], is one of the most verified patterns in both the biological and physical sciences [44]. It states that with respect to a non-negative stochastic variable *X*, the variance *V* = Var[*X*], or equivalently $\sigma^{2}$, is a power function of its mean μ=E[X]:

(6) $$V=a\mu^{b}$$

where a and b are both positive constants [45], in particular *b* is called the scaling exponent. It is titled by Taylor [46] who was the first one who proposed it after he surveyed some classical population such as virus lesions, macro zoo-plankton, worms, and symphylids in the soil, on the plants, and in the air, mites on leaves, ticks on sheep, and fish in the sea. Given its generality, Taylor’s law can be applied to many fields of study. In ecology, the random variable of interest is generally the size or density N of a censused population and Taylor’s Law can arise in time or in space [47]. In addition, it was used to predict a decrease in the population abundance variation along with a decrease in population density [48], the relationship between prevalence and abundance given by the epidemiological model developed for macroparasites providing a a measure of aggregation of parasites within their hosts [49] the spatial distribution of plants and animals per unit area [50]. Originally applied in ecology, the use of Equation (6) was spread in other sciences such as mathematics in order to show that the variance and the mean of the primes do not exceed a real number sufficiently large obeying Taylor’s law asymptotically [51]. Therefore, the present work aims to validate a theory according to Taylor’s law approach for turbulent flow field and, more precisely, in the context of vortex shedding. Our approach is based on a direct scaling since analyses do not fit any specific model of v(t). This empirical approach is well-known in literature, see for example [52] and in other contexts as to estimate the rate of sea level rise at some selected tide gauges around the world [53], in porous media [54,55], and in the framework of fluid mechanics [56,57].

## 3. Experimental Results and Discussions

### 3.1. Correlation Time

In the preliminary phase, our experimental analysis aims to find the aggregation time of the measures.

Hence, before analyzing the aggregation processes, it was necessary to understand what time-scale Δt considers to find a fluctuation scaling and also be useful for Strouhal measures. For this purpose, the *autocorrelation coefficient* R(τ), estimable for any stochastic process according to Equation (7), was helpful [58]:

(7) $$R\left(\tau \right)=\frac{C(\tau )}{\sqrt{Var\left[\upsilon \left(t\right)\right]Var\left[\upsilon \left(t^{\prime }\right)\right]}}$$

we recall that the autocovariance C(τ) is a function corresponding to the covariance, Cov, of the process with itself to pairs of time points, *t* and $t^{\prime }$, defined as follows [58]:

(8) $$C\left(\tau \right)=C\left(t,t^{\prime }\right)=Cov\left(\upsilon \left(t\right),\upsilon \left(t^{\prime }\right)\right)$$

with time-lag $\tau =t^{\prime }-t$. Obviously, it must be −1 ≤ R(τ) ≤ 1. For processes arising in turbulent flows, we expect the autocorrelation R(τ) to diminish as the lag time τ increases. Usually, it decreases sufficiently rapidly that the integral of Equation (7), called integral time-scale of the process, converges [59]:

(9) $$\Gamma =\int_{0}^{+\infty }R\left(\tau \right)\;d\tau$$

**Figure 7:**
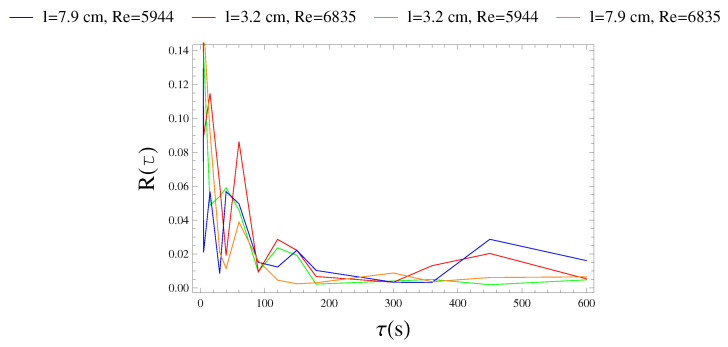
Development of the autocorrelation function over aggregation time for each configuration.

From a physical point of view, Γ represents the time interval necessary for the process to lose memory of its initial state [59]. The graphs in Figure 7 show the trend of auto-correlation function referred to fluctuating signal, with reference to four recordings for a total of 30 min corresponding to the two length cantilever (*l* = 3.2 cm and *l* = 7.9 cm) at two different flow fields (Re = 5944 and Re = 683). The auto-correlation function reaches zero value already at a time-scale of 3 min. Only the configuration characterized by *l* = 7.9 cm and Re=5944 is equal to zero at a time-scale of 5 min. Hence, voltage fluctuations behave similarly to a turbulent structure. For this reason, our analysis must concern aggregation processes with a time-scale lower than 3 min.

### 3.2. Vortex Shedding Measures and Relationship Voltage-Pressure

**Figure 8:**
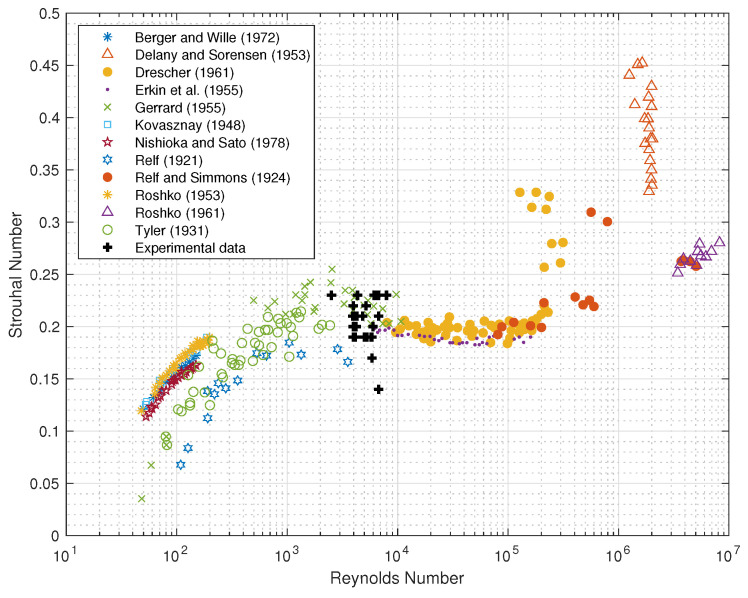
Strouhal number–Reynolds number relationship for circular cylinders taken from [5], the black crosses represent our experimental data.

In order to study the vortex shedding phenomenon, we defined Strouhal number St considering in the Equation (2) the value of the frequency of vortex shedding $f_{r}$ equal to the peak frequency $f_{peak}$ obtained applying the Fast Fourier Transform (FFT) to the time history of the fluctuating voltage v(t) recorded by the device (see Table 2), being careful to delete the influence due to noise. For this purpose, a lowpass filter with a cutoff frequency $\omega_{c}$ has been applied to the signal. By making a comparison with the values of natural frequencies $f_{1}$ shown in Table 1, we can state that the the danger due to the phenomenon of resonance is averted. Experimental data are plotted in the plane St−Re in which they are collected points corresponding to several experiments well-known in literature (see Figure 8). As shown in Figure 8, our data are in the region of turbulent vortex trail and also called laminar boundary layer on cylinder [40,60], also called subcritical regime by Fey et al. (1998) [37]. Based on these results, we can state that the values of the peak frequency $f_{peak}$ of the signal of the voltage fluctuations are comparable with the value of frequency of vortex shedding $f_{r}$ derived taking in the Equation (2) the Strouhal number St=0.21 as should be for the range of Reynolds number analyzed.

Regarding the relationship between the pressure difference Δp on each side of the flexible structure, provoked by the fluid motion, and the peak to peak voltage $v_{peak}$ of the piezoelectric material, shown in Equation (5), a mean value of constant *K* was evaluated. The mechanical and electrical properties of cantilever are listed as follows: the piezoelectric strain constant $d_{31}$ = 2 $\cdot 10^{-12}$ m V${}^{-1}$, the relative dielectric constant $e_{r}$ = 10.5, the dielectric constant in vacuum $e_{0}$ = 8.85 $\cdot 10^{-12}$ F m${}^{-1}$, the thickness of piezoelectric material $T_{pzt}$ = 1 μm, the cantilever lengths *l* are shown in Table 2. Numerical results highlighted a mean value of $K=\frac{1}{2}\cdot 10^{-5}$, such a value belongs to range between 0.09$\;\cdot \;10^{-5}$ and 1.41$\;\cdot \;10^{-5}$. Therefore, based on these last considerations, if we indicate with *Z* the following expression:

(10) $$Z=\frac{d_{31}}{e_{0}e_{r}}\frac{l^{2}}{T_{pzt}}$$

we can determine the following direct relationship between the peak to peak voltage $v_{peak}$ and the pressure difference Δp as follows:

(11) $$v_{peak}=KZ\Delta p$$

where the product K·Z is in the average equal to $4\cdot 10^{-6}$. Hence, it is possible to state the following approximated empirical formulae:

(12) $$v_{peak}∼4\cdot 10^{-3}\Delta p$$

with $v_{peak}$ expressed in mV and Δp in Pa.

### 3.3. Taylor’s Law Analysis

**Figure 9:**
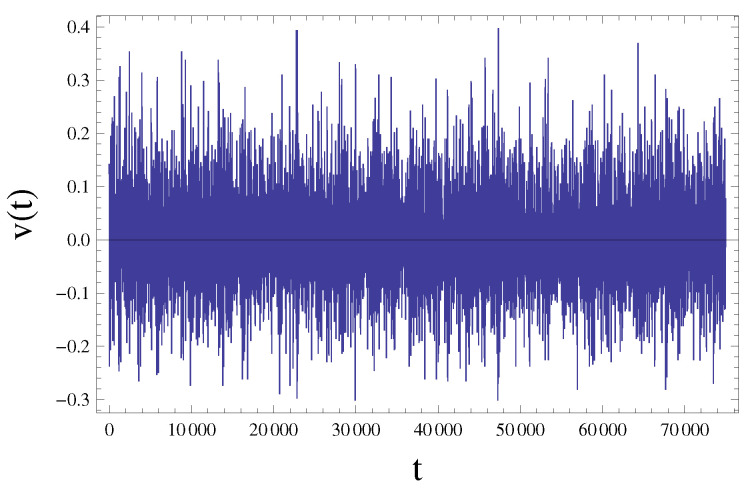
Time history of the voltage fluctuations v(t).

**Figure 10:**
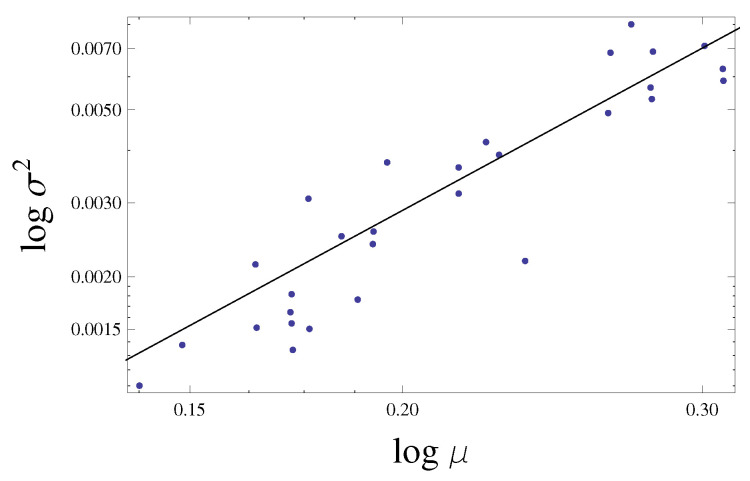
Taylor’s law in the log–log plane at a time-scale equal to one minute for the first configuration.

Starting from the signal of voltage fluctuations v(t) shown in Figure 9 and recorded by the oscilloscope, all values were shifted up, in order to have strictly positive voltages, according to Taylor’s law (Equation (6)). Given a period of fluctuating signal equal to 30 min for the configurations discussed in Section 3.1, we consider the following time-scales in seconds Δt = 5, 15, 30, 40, 60, 90, 150, 180. For each time-scale, the mean and variance were computed and plotted in a bi-logarithmic plane and through a numerical non-linear fitting procedure with $Mathematica^{©}$, an interpolating law of Equation (6) has been found from the following relationship:

(13) logV=loga+blogμ

that represents a linear law in the log–log plane (see Figure 10).

Once known the parameters of Taylor’s law *a* and *b* (Table 3), it was evaluated the correlation coefficient $r^{2}$ that represents a measure of fitting degree of the model to data points.

The values of $r^{2}$ relative to each time aggregation range for all the configurations are reported in the Table 4.

**Figure 11:**
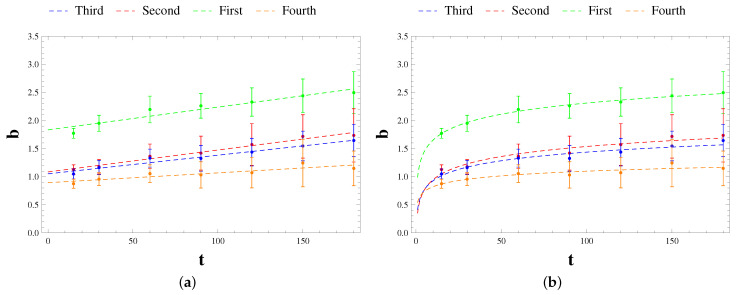
Laws of variation of scaling exponent over the aggregation time: linear law (**a**) and logarithmic law (**b**).

From the values of $r^{2}$ in the Table 4, it appears that regardless of the configuration and time-scale considered, the correlation between the experimental data and the fitting Taylor’s law is strong, since $r^{2}$ is very close to one for all analyses carried out. By Table 3, the minimum value of scaling exponent *b* is 0.875, assumed by the fourth configuration at the time-scale equal to 15 s, instead the maximum value is 2.496 reached by the first configuration at a time-scale of 3 min. The predominant range is the one between values 1 and 2, typical for the geographical population with relatively moderate degrees of aggregation [61]. The only two configurations characterized by scaling exponents outside this range are the first and the fourth configuration. The scaling law referring to the first configuration presents values of scaling exponent higher than other configurations. This is reasonable for the highly fluctuating time history of the signal (see Figure 9a) probably due to the mechanical characteristics of material such as the increase of stiffness inducted by the adapt to fatigue [29]. Instead, the fourth configuration is characterized by the lowest values of scaling exponent since the fluctuating signal is almost homogeneous (see Figure 9d). From a general point of view, the scale exponent *b* increases with the aggregation time Δt, no matter the configuration. According to the least squares method, both linear (Figure 11a) and logarithmic law (Figure 11b) have been found in order to maximize the correlation coefficient $r^{2}$, consequently it has pointed out that the logarithmic law is the best fitting line given that the scale exponent grows slightly with the aggregation time.

With the reference to the earlier cases of application of Taylor’s law, a logarithmic trend of scaling exponent on aggregation time has also been found by [62] investigating the endogenous and exogenous dynamics of 1354 stocks traded in the Chinese stock market. In addition, analyzing the time series of trading volume of 22 liquid stocks traded on Shenzen Stock Exchange in 2003, Mu et al. [63] reached the same outcome. Based on the previous results, we can state a general variation law of the scaling exponent over time-scale:

(14) $$b\left(\Delta t\right)=b_{0}+k\log\Delta t$$

The values of $b_{0}$ and *k* related to the configurations are summarized in Table 5, by which we can deduce that $b_{0}$ varies strongly with the configuration considered, hence it probably depends on the flag length and height of the open channel, while *k* belongs to the narrow range between 0.12 and 0.28.

**Figure 12:**
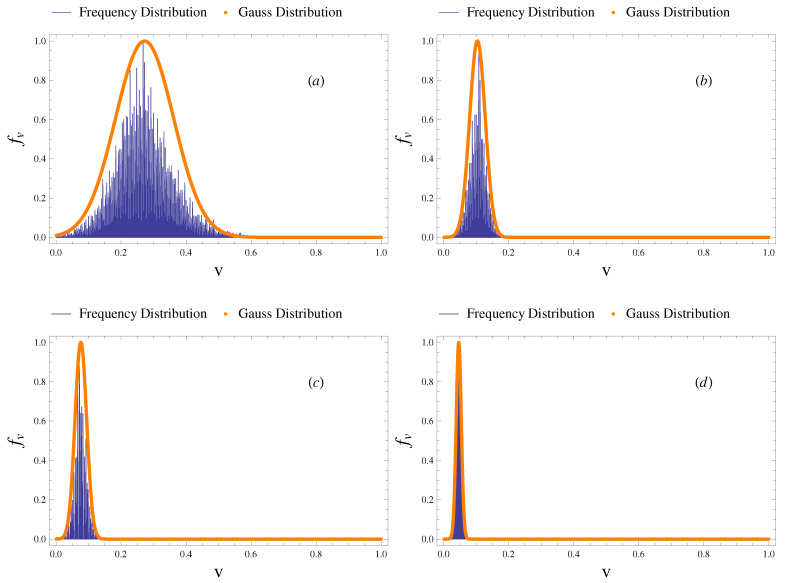
Comparison between the observed frequency distribution and the Gaussian probability density function.

At this point, it is important to understand how the fluctuations v(t) are distributed. If we plot in a plane (Figure 12), whose horizontal axis represents the values of voltage v(t) and the vertical axis the frequency distribution $f_{V}$ with a width bin equal to 0.001 volts, we obtain the histogram of frequency from which we can make some considerations for each configuration analyzed.

For example, the first configuration (see Figure 12a), given its shape, is characterized by a considerable degree of uncertainty. This means that the distribution has a high standard deviation according to results listed in the Table 3. In the second and third configurations, we notice the presence of skewness, with a tail on the right side in the second (see Figure 12b) and a tail on the left side in the third (see Figure 12c). The fourth configuration (see Figure 12d) has the lowest standard deviation according to the highly homogeneous shape of the fluctuating signal. Then, a comparison between the histogram of frequency and some theoretical probability density function has been carried out. Anyway, by means of the Method of Moments, it appears that the probability density function with more likehoods to the real distributions of the voltage fluctuations v(t) is the Gaussian one. This result is in agreement with the experimental evidence and numerical simulations as well-known in literature about the distribution of the fluctuating component of velocity $u^{\prime }\left(t\right)$ [58,59,64,65,66]. Therefore, based on this encouraging preliminary survey, several geometric analyses in the channel flume will be carried out in order to validate this sensor in other ranges of Strouhal and Reynolds numbers and on obstacles of other shapes.

## 4. Conclusions

In the present work, a thin films-based piezoelectric device, approximated to a cantilevered bending, is proposed for providing useful information for the flow field in the open channel, especially in the context of vortex shedding. Several experiments were performed in a channel flume site at the EUMER Lab at University of Salento for a specific range of the $Re=10^{3}$ order. As a first step, based on the analysis shown in Section 3.2, it appears that the peak frequency of the voltage fluctuations, recorded by the device and filtered by the influence of noise, is close to the frequency of vortex shedding for the flow field studied. Based on these results, we can state that the flapping device could be helpful to determine Strouhal number St, alternatively to the methods mentioned in Section 1. Then, a direct scaling analysis of the voltage fluctuations has been carried out. It has been estimated, for each analyzed configuration, in Section 3.1, the autocorrelation coefficient that shows an ergodic behavior of the signal and the time-scale within which turbulent structure is coherent. Consequently, it has been possible to demonstrate that fluctuations of the flapping device, in terms of voltage, respond to Taylor’s law, applied in several scientific and technical contexts, with the scaling exponents convergent in a particular range between 0.8 and 2.5, as seen in Section 3.3. Moreover, a constant $K=\frac{1}{2}\cdot 10^{-5}$ has been found in a relationship between the voltage and difference of pressure from each side of the flag (Equation (5)). Since the difference of pressure from each side of the flag is related to the rotational flow velocity of the whirls $u_{r}$ (Equation (4)), we have reason to believe that also vortex shedding phenomenon and consequently the rotational flow velocity of the whirls $u_{r}$ follow a scaling law (v(t)∼Δp∼$u_{r}$), as shown in the Equation (12). This consideration is enforced by a comparison between the frequency distribution of voltages and some theoretical probability density functions (see Figure 12), that allows us to state that the maximum similarity is reached with the Gaussian distribution. Further investigations are being planned for validating other orders of Reynolds number, also on obstacles of other shape, in order to find out if the scaling behavior of Taylor’s law could be applicable as well. Future challenges could consist of applying Taylor’s law to the velocity fluctuations, once the velocity cross-distribution in the open channel is known.

## Figures and Tables

**Table 1:** Values of natural frequency of vibration, $f_{1}$, of the cantilever for the several length *l*.

*l* [cm]	3.20	3.50	4.00	7.90
$f_{1}$ [Hz]	8.40	7.38	5.40	1.10

**Table 2:** Values of peak frequency $f_{r}$ of the voltage fluctuations v(t) and corresponding Strouhal number St for the different analyzed configurations characterized by lenght of flag *l*, heigth of flow *h* in the open channel, bulk velocity *u* and Reynolds number Re.

*l*	*H*	*u*	Re	$f_{r}$	St
(cm)	(cm)	(m s${}^{-1})$	(-)	(s${}^{-1})$	(-)
3.2	4.6	0.293	5859	2.50	0.17
3.2	4.0	0.337	6738	2.35	0.14
3.5	6.9	0.197	3934	2.09	0.21
3.5	6.8	0.198	3963	2.20	0.22
3.5	6.8	0.198	3934	1.93	0.19
3.5	6.7	0.201	4022	2.11	0.21
3.5	6.7	0.201	4022	2.12	0.21
3.5	6.6	0.204	4083	1.98	0.19
3.5	6.5	0.207	4146	2.05	0.20
3.5	6.5	0.207	4146	2.00	0.19
3.5	6.4	0.211	4211	2.13	0.20
3.5	6.3	0.214	4278	2.22	0.21
3.5	6.2	0.217	4347	2.45	0.23
3.5	6.2	0.217	4347	2.45	0.23
4.0	10.7	0.126	2519	1.45	0.23
4.0	6.8	0.198	3963	2.01	0.20
4.0	5.6	0.241	4813	2.54	0.21
4.0	5.4	0.250	4991	2.33	0.19
4.0	5.2	0.259	5183	2.86	0.22
4.0	5.1	0.264	5284	2.54	0.19
4.0	4.7	0.287	5734	2.72	0.19
4.0	4.5	0.299	5989	3.01	0.20
4.0	4.4	0.306	6125	3.50	0.23
4.0	4.3	0.313	6167	3.67	0.23
4.0	4.0	0.337	6738	3.90	0.23
4.0	3.4	0.396	7926	4.54	0.23
7.9	4.6	0.293	5859	2.71	0.19
7.9	4.0	0.337	6738	3.54	0.21

**Table 3:** Values of scaling exponent of Taylor’s law *b* for each configuration at several aggregation time ranges.

		b		
Δt	First	Second	Third	Fourth
(s)	l=3.2 cm	l=3.2 cm	l=7.9 cm	l=7.9 cm
	Re = 5944	Re = 6835	Re = 5944	Re = 6835
15	1.773	1.128	1.049	0.875
30	1.951	1.177	1.162	0.957
60	2.197	1.363	1.331	1.055
90	2.262	1.420	1.326	1.031
120	2.330	1.572	1.438	1.071
150	2.440	1,717	1.547	1.244
180	2.496	1.735	1.644	1.148

**Table 4:** Values of $r^{2}$ related to previous fitting laws for each configuration at several aggregation time ranges.

		$r^{2}$		
Δt	**First**	**Second**	**Third**	**Fourth**
**(s)**	l **= 3.2 cm**	l **= 3.2 cm**	l **= 7.9 cm**	l **= 7.9 cm**
	Re=5944	Re=6835	Re=5944	Re=6835
15	0.953	0.964	0.971	0.970
30	0.952	0.967	0.978	0.975
60	0.952	0.964	0.985	0.978
90	0.975	0.964	0.983	0.978
120	0.977	0.966	0.985	0.977
150	0.977	0.973	0.989	0.973
180	0.974	0.971	0.991	0.981

**Table 5:** Values of $b_{0}$ and *k* for the logarithmic laws.

	First	Second	Third	Fourth
$b_{0}$	0.989	0.353	0.416	0.544
*k*	0.287	0.257	0.222	0.120
